# Receptor-Targeted Photodynamic Therapy of Glucagon-Like Peptide 1 Receptor–Positive Lesions

**DOI:** 10.2967/jnumed.119.238998

**Published:** 2020-11

**Authors:** Marti Boss, Desiree Bos, Cathelijne Frielink, Gerwin Sandker, Patricia Bronkhorst, Sanne A.M. van Lith, Maarten Brom, Mijke Buitinga, Martin Gotthardt

**Affiliations:** Department of Radiology and Nuclear Medicine, Radboud University Medical Center, Nijmegen, The Netherlands

**Keywords:** glucagon-like peptide 1 receptor, exendin, photodynamic therapy, hyperinsulinemic hypoglycemia

## Abstract

Treatment of hyperinsulinemic hypoglycemia is challenging. Surgical treatment of insulinomas and focal lesions in congenital hyperinsulinism is invasive and carries major risks of morbidity. Medication to treat nesidioblastosis and diffuse congenital hyperinsulinism has varying efficacy and causes significant side effects. Here, we describe a novel method for therapy of hyperinsulinemic hyperglycemia, highly selectively killing β-cells by receptor-targeted photodynamic therapy (rtPDT) with exendin-4-IRDye700DX, targeting the glucagon-like peptide 1 receptor (GLP-1R). **Methods:** A competitive binding assay was performed using Chinese hamster lung (CHL) cells transfected with the GLP-1R. The efficacy and specificity of rtPDT with exendin-4-IRDye700DX were examined in vitro in cells with different levels of GLP-1R expression. Tracer biodistribution was determined in BALB/c nude mice bearing subcutaneous CHL-GLP-1R xenografts. Induction of cellular damage and the effect on tumor growth were analyzed to determine treatment efficacy. **Results:** Exendin-4-IRDye700DX has a high affinity for the GLP-1R, with a half-maximal inhibitory concentration of 6.3 nM. rtPDT caused significant specific phototoxicity in GLP-1R–positive cells (2.3% ± 0.8% and 2.7% ± 0.3% remaining cell viability in CHL-GLP-1R and INS-1 cells, respectively). The tracer accumulates dose-dependently in GLP-1R–positive tumors. In vivo, rtPDT induces cellular damage in tumors, shown by strong expression of cleaved caspase-3, and leads to a prolonged median survival of the mice (36.5 vs. 22.5 d, respectively; *P* < 0.05). **Conclusion:** These data show in vitro as well as in vivo evidence of the potency of rtPDT using exendin-4-IRDye700DX. This approach might in the future provide a new, minimally invasive, highly specific treatment method for hyperinsulinemic hypoglycemia.

Insulin production by pancreatic β-cells is usually a well-regulated process. However, uncontrolled overproduction of insulin can arise, in most cases as a result of insulin-producing lesions. Such lesions cause major clinical symptoms, and treatment can be challenging. In adults, these lesions manifest in endogenous adult hyperinsulinemic hypoglycemia, most often caused by an insulinoma, which is an insulin-producing neuroendocrine tumor arising from pancreatic β-cells (1). In 0.5%–5% of cases, adult hyperinsulinemic hypoglycemia is caused by nesidioblastosis, characterized by proliferation of abnormal β-cells throughout the pancreas (2). In neonates, the most common cause of persistent hyperinsulinism is congenital hyperinsulinism (CHI) (3). In diffuse CHI there is diffuse involvement of the pancreatic β-cells, whereas in focal CHI the disease is caused by focal adenomatous islet cell hyperplasia (4). Episodic hypoglycemia due to endogenous hyperinsulinism causes neuroglycopenic and autonomic symptoms. Prolonged hypoglycemia may lead to seizures, loss of consciousness, permanent brain damage, or brain death (5).

Insulinomas and focal CHI can be cured by surgical removal of the lesion (3,6). Enucleation is possible in cases of superficially localized lesions with sufficient distance from the pancreatic duct (2–3 mm). Otherwise, a more extensive surgical procedure such as partial or distal pancreatectomy may be required. Although such procedures can often be performed laparoscopically (7,8), they remain challenging and may carry major risks of morbidity (9,10). The only surgical treatment option for patients with nesidioblastosis and diffuse CHI not responding to medication is partial pancreatectomy. Even after such an invasive procedure, hypoglycemic episodes often persist, requiring continued treatment with medication and, in certain cases of CHI, total pancreatectomy (2,4).

Because of these challenges, a novel, preferably minimally invasive treatment option for hyperinsulinemic hypoglycemia in adults, as well as in children, is warranted. In this study, we assessed the feasibility of specific ablation of insulin-producing cells with interstitial photodynamic therapy (PDT). PDT is based on inducing cell death by irradiation of a light-sensitive molecule, or photosensitizer). The photosensitizer absorbs photons and is transferred to a higher-energy state. By transfer of energy from the activated photosensitizer to the oxygen in the surrounding tissue, reactive oxygen species are produced, which can cause cellular damage (11). To ensure efficient and specific delivery of the photosensitizer to the target tissue, the photosensitizer is coupled to a tumor-specific targeting moiety (12).

An attractive targeting moiety for receptor-targeted PDT (rtPDT) of insulin-producing cells is exendin-4. This peptide is a stable analog of the hormone glucagon-like peptide 1 (GLP-1). It specifically binds to the GLP-1 receptor (GLP-1R), which is expressed on pancreatic β-cells and at high levels in nearly 100% of benign insulinomas (13). GLP-1R imaging using ^111^In- and ^68^Ga-labeled exendin-4 has been shown to be a successful preoperative imaging technique for insulinomas (14–16) and is also under investigation in CHI (NCT03768518; clinicaltrials.gov).

We have developed an approach for rtPDT of insulin-producing lesions using the peptide exendin-4 coupled to the photosensitizer IRDye700DX (LI-COR Biosciences). We hypothesize that this novel method will allow specific cell killing of GLP-1R–positive cells.

## MATERIALS AND METHODS

### Reagents

Exendin-4-IRDye700DX was supplied by piCHEM. IRDye700DX NHS ester was obtained from LI-COR Biosciences. IRDye700DX absorbs and emits light in the near-infrared (NIR) range and has a higher extinction coefficient (2.1 × 10^5^ M^−1^cm^−1^ at 689 nm) than non-NIR photosensitizers (12,17). The N-ε-amino group of lysine at position 40 was site-specifically modified during solid-phase peptide synthesis with a mercaptopropionic acid, releasing an unprotected exendin-4 with a free thiol function after triisopropylsilane cleavage. IRDye700DX was modified with a maleimide, and coupling to exendin-4 was performed using a thiol reactive crosslinking approach. The purity was more than 90%. Stock solutions of exendin-4-IRDye700DX were prepared in phosphate-buffered saline (PBS). The structure and amino acid sequence of the tracer are shown in Supplemental Figure 1 (supplemental materials are available at http://jnm.snmjournals.org). The absorbance and emission spectra of exendin-4-IRDye700DX are shown in Supplemental Figure 2.

### Cell Culture

Chinese hamster lung (CHL) cells stably transfected with the GLP-1R (18) were cultured in Dulbecco modified Eagle medium with 4.5 g/L d-glucose and GlutaMAX (Invitrogen), supplemented with 10% fetal calf serum, 100 IU/mL penicillin G, 10 mg/mL streptomycin, 1 mM sodium pyruvate, 0.1 mM nonessential amino acids, and 0.3 mg/mL G418 geneticin. The rat insulinoma cell line INS-1 was cultured in RPMI 1640 medium, supplemented with 10% fetal calf serum, 100 IU/mL penicillin G, 10 mg/mL streptomycin, 2 mmol/L l-glutamine, 1 mmol/L pyruvate, 10 mmol/L 4-(2-hydroxyethyl)-1-piperazineethanesulfonic acid, and 50 μmol/L 2-mercaptoethanol. The human pancreatic tumor cell line PANC-1 was cultured in RPMI 1640 medium supplemented with 10% fetal calf serum, 100 IU/mL penicillin G, 10 mg/mL streptomycin, and 2 mmol/L l-glutamine.

### Competitive Binding Assay

The half-maximal inhibitory concentrations of exendin-4-IRDye700DX and unlabeled exendin, as a reference, were determined using CHL-GLP-1R cells as described previously (19,20). One million cells per well were grown overnight in 6-well plates. The cells were washed twice with PBS and incubated for 4 h on ice with 50,000 cpm of ^111^In-labeled exendin in the presence of increasing concentrations of exendin-4-IRDye700DX (0.1–300 nM). The cells were then washed with PBS, solubilized with 2 mL of NaOH, and collected, and the cell-associated activity was measured in a γ-counter (Wizard 2480; PerkinElmer).

### In Vitro rtPDT

CHL-GLP-1R cells, INS-1 cells, and PANC-1 cells were seeded into 24-well plates (Thermo Scientific) (150,000 cells per well) and grown overnight. Medium was replaced by binding buffer (medium with 0.1% bovine serum albumin [w/v] [BSA]) with exendin-4-IRDye700DX (300 nM for CHL-GLP-1R cells and 400 nM for INS-1 and PANC-1 cells [concentrations based on optimization experiments]). As a control, cells incubated with binding buffer only were used. Separate wells were incubated with an excess (15 μM for CHL-GLP-1R cells and 20 μM for INS-1 and PANC-1 cells) of unlabeled exendin-4 together with exendin-4-IRDye700DX. After incubation at 37°C (CHL-GLP-1R cells, 4 h; INS-1 and PANC-1 cells, 24 h), cells were washed with binding buffer. Subsequently, cells were irradiated with an NIR light–emitting diode (LED) (21) (emission wavelength, 670–710 nm; forward voltage, 2.6 V; power output, 490 mW) using 126 individual LED bulbs ensuring homogeneous illumination (21). CHL-GLP-1R cells were irradiated at 90 J/cm^2^ (over 6 min). INS-1 and PANC-1 cells were irradiated at 150 J/cm^2^ (over 10 min). Cells incubated with exendin-4-IRDye700DX that were not irradiated were included as a control. All experiments were performed in triplicate.

Four hours after irradiation, during which the cells were kept at 37°C and 5% CO_2_, the adenosine triphosphate content as a measure of cell viability was determined using a CellTiter-Glo luminescent assay (Promega Benelux) according to the instructions of the manufacturer. Luminescence was measured using a Tecan Infinite M200 PRO plate reader (PerkinElmer). The adenosine triphosphate content as a measure of cell viability was expressed as a percentage, determined by comparing the luminescent signal with the signal from untreated cells, which were considered 100% viable.

Additionally, a coculture of INS-1 and PANC-1 cells was plated in 24-well plates (70,000 and 40,000 cells per well, respectively). Before seeding, INS-1 cells were labeled with the fluorescent dye DiO and PANC-1 cells with DiD dye according to the manufacturer’s protocol (Life Technologies, Thermo Fisher Scientific). The cells were grown overnight and then incubated with 400 nM exendin-4-IRDye700DX in binding buffer or binding buffer alone for 24 h at 37°C and 5% CO_2_. Subsequently, the cells were irradiated with 150 J/cm^2^ of NIR light. After 4 h, the cells were incubated with 1 μg/mL propidium iodide (Thermo Fisher Scientific) in PBS for 15 min at room temperature. The cells were visualized using an EVOS microscope (Thermo Fisher Scientific).

### Animal Tumor Model

Female BALB/c nude mice (Janvier), 6–8 wk old, were housed in individually ventilated cages (6 mice per cage) under nonsterile conditions with ad libitum access to chlorophyll-free animal chow and water. CHL-GLP-1R cells (5 × 10^6^ cells per mouse in 200 μL of Dulbecco modified Eagle medium with 4.5 g/L d-glucose and GlutaMAX) were injected subcutaneously on the right flank of the mice.

### In Vivo Biodistribution

Female BALB/c nude mice with CHL-GLP-1R xenografts were injected intravenously with exendin-4-IRDye700DX in 200 μL of PBS with 0.5% BSA (5 mice per group; 1, 3, and 10 μg of exendin-4-IRDye700DX). Four mice were injected with only PBS with 0.5% BSA. After 4 h, the mice were killed by CO_2_ asphyxiation, and the tumor and organs were removed and collected in MagNA Lyser tubes (F. Hoffmann-La Roche Ltd.). Radioimmunoprecipitation assay lysis buffer (500 μL; 50 mM (hydroxymethyl)aminomethane-hydrochloride, pH 7.4, with 150 mM NaCl, 1 mM ethylenediaminetetraacetic acid, 1% Triton-X-100 (Dow Chemical Co.), and 1% sodium dodecyl sulfate) was added to each tube. Organs were homogenized using a MagNA Lyser (F. Hoffmann-La Roche Ltd.) with repeated cycles of 6,000 rpm for 25 s with cooling on ice for 1 min between cycles. Organ homogenates of the control mice (injected only with PBS with 0.5% BSA) were used to create standard curves for exendin-4-IRDye700DX for each organ. A 100-μL volume of homogenates was transferred in triplicate to a black flat-bottom 96-well plate, and fluorescence intensity was measured using a Tecan Infinite M200 PRO plate reader (excitation wavelength, 620 nm; emission wavelength, 700 nm). Standard curves and tracer uptake were calculated using Microsoft Office Excel 2007.

### rtPDT In Vivo: Immunohistochemistry

Female BALB/c nude mice with subcutaneous GLP-1R–positive xenografts (8 mice per group) were injected intravenously with 30 μg of exendin-4-IRDye700DX in 200 μL of PBS with 0.5% BSA or in 200 μL of PBS with 0.5% BSA only and, after 4 h, exposed to 100 J/cm^2^ NIR LED light. One group was treated only with exendin-4-IRDye700DX without NIR light exposure. At 2 or 24 h after NIR light exposure, the mice were killed by CO_2_ asphyxiation. Tumors were harvested, fixated in 4% buffered formalin, embedded in paraffin, and sectioned at a 4-μm thickness. Slices were deparaffinized with xylene and rehydrated in ethanol. Antigen was retrieved with 10 mM citrate, pH 6.0, in a PT-Module (Thermo Fisher Scientific) (10 min, 96°C). Endogenous peroxidase activity was quenched with 3% H_2_O_2_ for 10 min. Slices were incubated with 20% normal goat serum for 30 min and subsequently with rabbit-anticleaved caspase-3 (1:4,000 in PBS + 1% BSA, ASP175; Cell Signaling Technology) in a humidified chamber at 4°C overnight in the dark. Slides were then washed 3 times with 10 mM PBS and incubated with goat-antirabbit-biotin (1:200 in PBS + 1% BSA; Vector Laboratories) for 30 min at room temperature. After being washed with PBS, slides were incubated with Vectastain Elite ABC kit (Vector Laboratories) for 30 min at room temperature. The bound antibodies were visualized using diaminobenzene (Bright-DAB, catalog no. BS04 Immunologic; VWR). Slides were counterstained with 3-times-diluted hematoxylin (Klinipath) for 5 s and mounted with a cover slip (Permount; Fisher Scientific).

The immunohistochemical staining was independently analyzed by 2 masked observers. Scores were allocated to each slide following an ordinal 6-point scale ranging from 0 to 5 (0, no staining; 1, very weak staining; 2, weak staining; 3, intermediate staining; 4, intense staining; 5, very intense staining). The scores of the 2 observers were averaged.

### rtPDT In Vivo: Survival

Female BALC/c nude mice with CHL-GLP-1R xenografts were randomized into 2 groups of 8 animals based on tumor size. When tumors were at least 30 mm^3^, the mice were injected intravenously with 30 μg of exendin-4-IRDye700DX in 200 μL of PBS with 0.5% BSA or PBS with 0.5% BSA only. After 4 h, the mice were exposed to 150 J/cm^2^ of NIR LED light under inhalation anesthesia (2.5% isoflurane mixed with 100% O_2_ (1 L/min)). The kidneys were protected from exposure by covering them with gauze and aluminum foil. Tumor diameters were measured by a masked observer 3 times per week in 3 dimensions using a caliper. The mice were euthanized by CO_2_ asphyxiation when tumor volume reached more than 1,000 mm^3^ (tumor volume was calculated by 1.25 × π × [([length + width + height]/6)^3^]). Overall survival was defined as the day that tumors reached a size of 1,000 mm^3^.

### Statistics

Statistical calculations were performed using Prism (version 5.03; GraphPad Software). Half-maximal inhibitory concentrations were calculated by fitting the data with nonlinear regression using a least-squares fit with Prism. In vitro cell viability after various treatments, assessed by a CellTiter-Glo assay, were compared by 2-way ANOVA with post hoc Bonferroni tests. Tracer uptake in various tumors was compared between the different injected doses by 1-way ANOVA.

Survival curves were compared by the log-rank (Mantel–Cox) test using Prism.

### Study Approval

All animal experiments were approved by the institutional Animal Welfare Committee of the Radboud University Medical Centre and were conducted in accordance with the guidelines of the Revised Dutch Act on Animal Experimentation.

## RESULTS

### Exendin-4-IRDye700DX Binds GLP-1R with High Affinity

**FIGURE 1.**
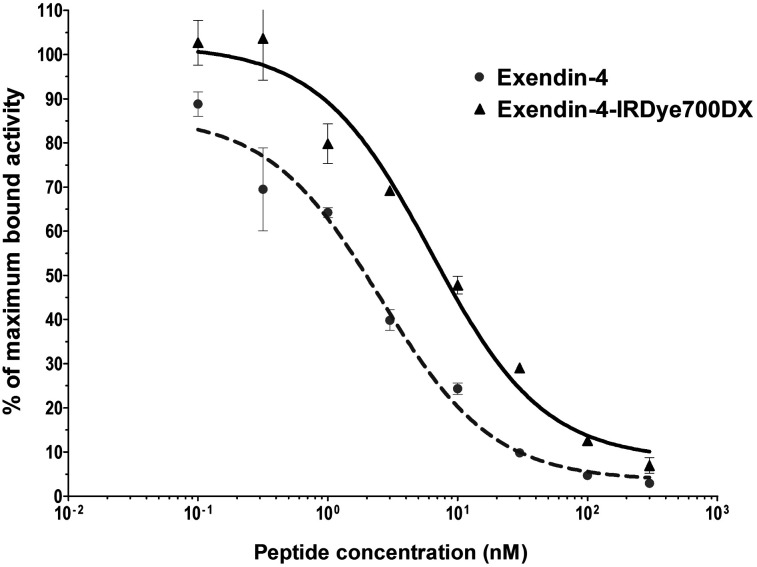
Competition binding assay (half-maximal inhibitory concentration) using CHL-GLP-1 cells of unlabeled exendin-4 and exendin-4-IRDye700DX. ^111^In-DTPA-exendin-4 was used as tracer.

The half-maximal inhibitory concentrations of exendin-4 and exendin-4-IRDye700DX were 2.54 nM (95% confidence interval, 1.32–4.90) and 6.25 nM (95% confidence interval, 3.07–12.74), respectively (Fig. 1). Although the binding affinity of the labeled peptide was significantly lower than that of the unlabeled peptide (*P* < 0.0001), it bound with a high affinity to the GLP-1R in the nanomolar range.

### In Vitro rtPDT with Exendin-4-IRDye700DX and NIR Light Causes Specific GLP-1R–Positive Cell Death

**FIGURE 2.**
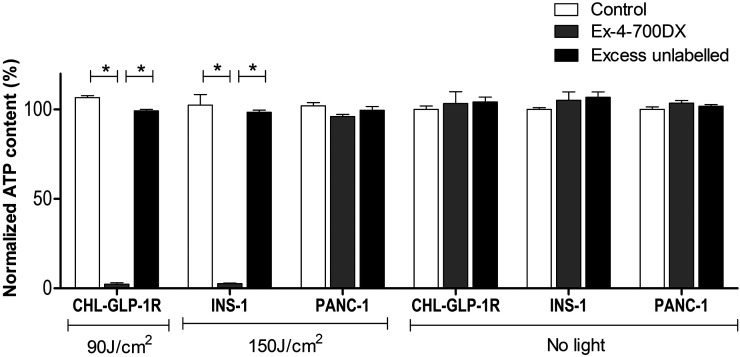
Adenosine triphosphate (ATP) content as measure of viability of CHL-GLP-1R cells, INS-1 cells, and PANC-1 cells after incubation with binding buffer (control), exendin-4-IRDye700DX, or exendin-4-IRDye-700DX combined with excess of unlabeled exendin-4 and with or without NIR light irradiation. Experiments were performed in triplicate. Data are presented as mean ± SD. *P < 0.001.

**FIGURE 3.**
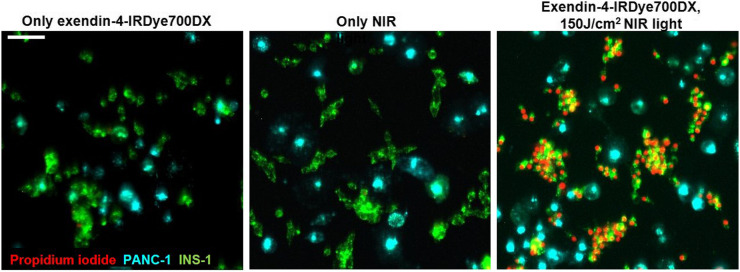
Fluorescence microscopy of INS-1 cells labeled with fluorescent dye DiO (green) and PANC-1 cells labeled with fluorescent dye DiD (cyan), cocultured and incubated with propidium iodide (red), after incubation of exendin-4-IRDye700DX or only binding buffer and with and without NIR irradiation with radiant exposure of 150 J/cm^2^. Scale bar denotes 100 μm.

rtPDT with exendin-4-IRDye700DX caused significant phototoxicity in cells with high GLP-1R expression (CHL-GLP-1R cells) and in the rat insulinoma cell line (INS-1 cells), with GLP-1R expression comparable to that in human insulinomas. The remaining cell viabilities were 2.3% ± 0.8% and 2.7% ± 0.3%, respectively (Fig. 2). In PANC-1 cells, no cellular phototoxicity was observed under these conditions (96.1% ± 1.2% viable cells). Coincubation with an excess of unlabeled exendin-4 abolished the phototoxic effect in CHL-GLP-1R cells and in INS-1 cells (99.3 ± 1.3 and 98.4% ± 2.1% cell viability, respectively). NIR light irradiation alone did not cause cellular phototoxicity in any of the cell types (106.6% ± 1.2%, 102.5% ± 5.9%, and 102.0% ± 1.8% viable cells in CHL-GLP-1R, INS-1, and PANC-1 cells, respectively). No dark toxicity of the tracer was observed (103.3% ± 6.7%, 105.2% ± 4.7%, and 103.6% ± 1.4% cell viability without irradiation in CHL-GLP-1R, INS-1, and PANC-1 cells, respectively). Incubation of a coculture of INS-1 and PANC-1 cells with exendin-4-IRDye700DX followed by irradiation specifically caused cell death in INS-1 cells, as shown by colocalization of the red and green nuclei (Fig. 3). Absence of postinjection signal on rtPDT indicated that exendin-4-IRDye700DX alone or NIR light alone did not cause cell death in either cell type.

### Exendin-4-IRDye700DX Accumulates in GLP-1R–Positive Tumors

**FIGURE 4.**
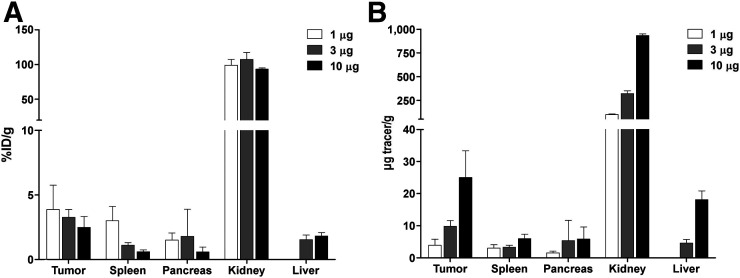
Biodistribution of exendin-4-IRDye700DX (1, 3, and 10 μg; 5 mice per group) in tumors, spleen, pancreas, kidneys, and liver of female BALB/c nude mice 4 h after tracer injection. (A) Relative uptake expressed as percentage injected dose (%ID) per gram of tissue. (B) Absolute uptake expressed as micrograms of exendin-4-IRDye700DX per gram of tissue.

Relative uptake of exendin-4-IRDye700DX in subcutaneous GLP-1R tumors in mice was 3.9% ± 1.9% injected dose/g for a 1-μg tracer dose and diminished slightly to 3.3% ± 0.6% for a 3-μg tracer dose and 2.5% ± 0.8% for a 10-μg tracer dose (*P* = 0.25) (Fig. 4). As a result, the absolute tumor uptake increased with increasing injected tracer doses to 25.0 μg/g with a 10-μg tracer injection. Uptake of exendin-4-IRDye700 was highest in the kidneys, because of renal clearance.

### In Vivo rtPDT Causes Cell Death in GLP-1R–Positive Tumors and Improves Survival

**FIGURE 5.**
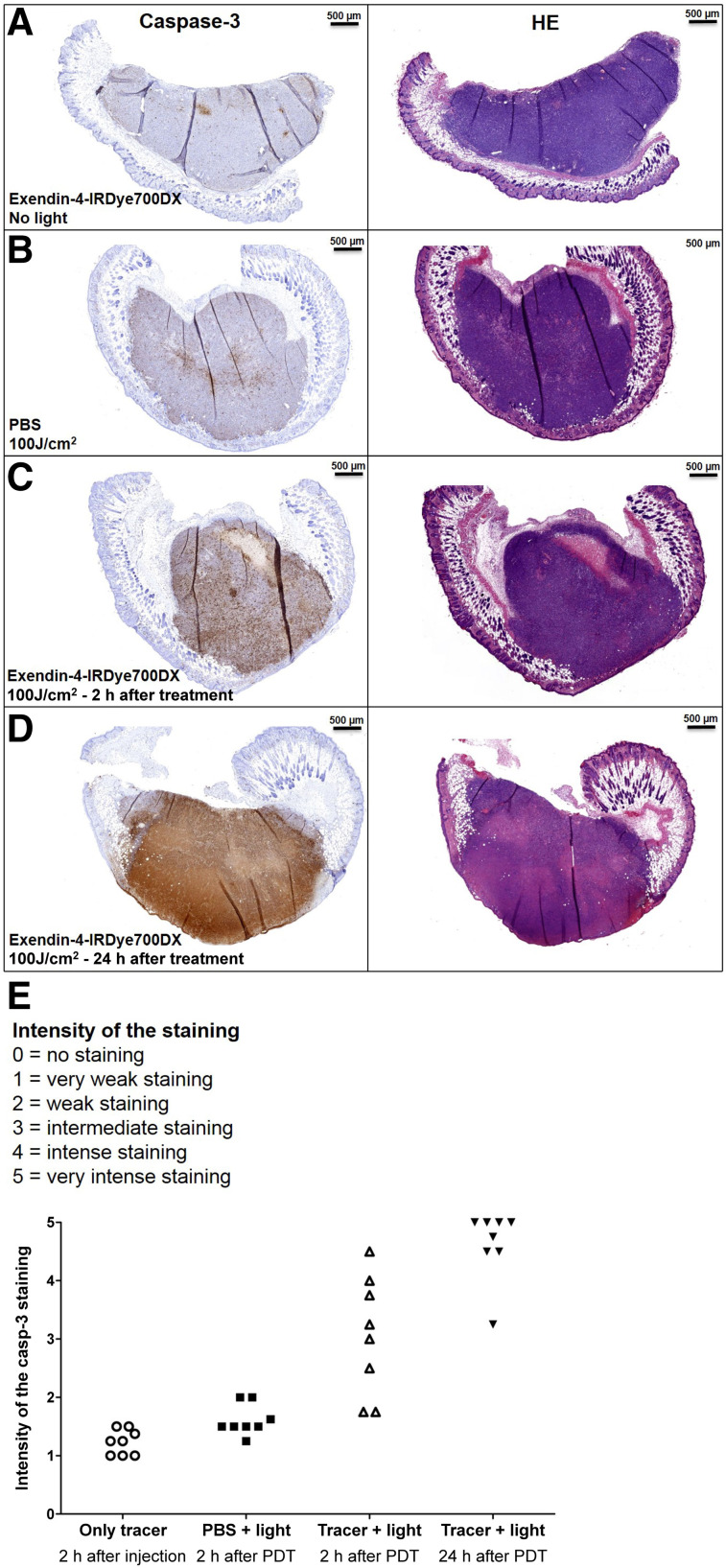
Representative examples of cleaved caspase-3 and hematoxylin and eosin (HE) staining of CHL-GLP-1R tumors. (A) Control tumors after intravenous administration of exendin-4-IRDye700DX. (B) Control tumors after only illumination. (C) Tumors after intravenous administration of exendin-4-IRDye700DX and illumination, dissected after 2 h. (D) Tumors after intravenous administration of exendin-4-IRDye700DX and illumination dissected after 24 h. (E) Intensity scores of capase-3 staining for tumor sections of all mice.

Analysis of the immunohistochemical staining revealed a low expression of cleaved caspase-3 in the control groups. In both treatment groups, the expression of cleaved caspase-3 was higher than in the control groups. Although the intensity of cleaved caspase-3 staining was variable at 2 h after treatment, the intensity of the staining was high and uniform in the tumors 24 h after treatment, showing a significant induction of apoptosis in the tumors. The expression of cleaved caspase-3 was slightly increased in the control group receiving only NIR light irradiation, showing that the light itself induces some cell death, most likely because of the heat produced by the LED light source (Fig. 5).

**FIGURE 6.**
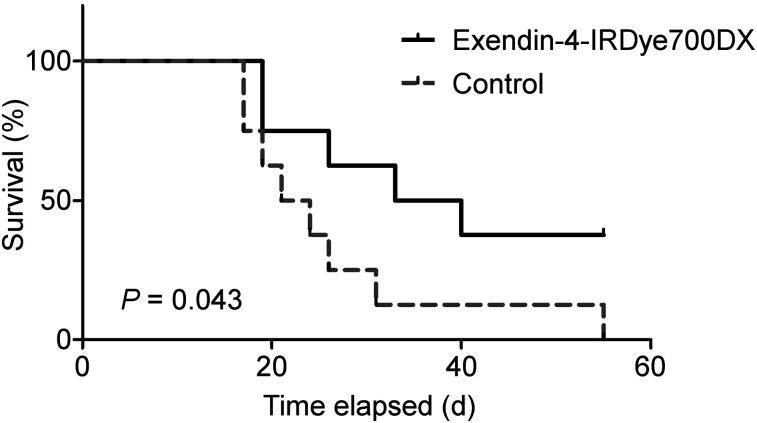
Kaplan–Meier plot of survival of BALB/c nude mice with GLP-1R–positive tumors after injection of 30 μg of exendin-4-IRDye700DX or PBS (control), followed by illumination with radiant exposure of 150 J/cm^2^.

At the start of the survival experiment, the sizes of the subcutaneous GLP-1R tumors were very variable, although mean tumor sizes were similar between the groups (161 ± 205 mm^3^ [range, 35–657 mm^3^] in the exendin-4-IRDye700DX group and 171 ± 144 mm^3^ [range, 36–480 mm^3^] in the control group. On light exposure, tumor growth was slower in the group that received exendin-4-IRDye700DX, leading to a significantly longer median survival in this group than in the control group (36.5 vs. 22.5 d, respectively; *P* < 0.05) (Fig. 6).

## DISCUSSION

Treatment of hyperinsulinemic hypoglycemia is challenging. To address this issue, a strategy that specifically destroys GLP-1R–positive cells with rtPDT was developed as an alternative treatment option for all forms of hyperinsulinemic hypoglycemia.

We showed the effectiveness of rtPDT with exendin-4-IRDye700DX in vitro and in vivo. The specific cytotoxic effect demonstrated that rtPDT with exendin-4-IRDye700DX might enable destruction of GLP-1R–positive lesions without damaging the surrounding pancreatic tissue.

To our knowledge, this is the first evidence of the effectiveness of a peptide-based agent for rtPDT in vivo. In the current development of tracers for rtPDT, the most widely used carrier molecules are monoclonal antibodies and nanoparticles, because of their slow clearance from the circulation and high uptake in target organs. A single previous study examining rtPDT using various targeting peptides was limited to in vitro studies and showed no efficient cytotoxic effect (22).

We believe that rtPDT with exendin-4-IRDye700DX has the potential to be used as a minimally invasive technique to destroy insulin-producing cells with minimal morbidity. On delivery of the tracer, NIR light can be administered interstitially using diffuser fibers placed into the target tissue. Using this method of so-called interstitial PDT, it is feasible to deliver light to deeply seeded lesions or tissues. Successful results of interstitial PDT have been obtained in, for example, prostate cancer (23), head and neck cancer (24), and, importantly, pancreatic tumors (25). An optimal treatment result depends on optimization of the number of light sources and of their specific placement and power output (26–28). With percutaneous delivery, areas up to 23 cm^2^ can be treated (29), making the technique suitable for treatment of CHI and nesidioblastosis. Alternatively, the less invasive endoscopic delivery of a fiber can be applied for treatment of small lesions, since a single fiber can be applied using this technique (30,31).

The data in this paper do not show 100% cell killing. Because these experiments were performed on an immunocompromised mouse model, they did not take into account the possible added effect on cell killing of the immune response elicited by PDT, as has been shown for other tumor types (32). Additionally, because of the minimal invasiveness of PDT, treatment can easily be repeated if hypoglycemia persists. Of interest, in a clinical situation, killing of enough cells to prevent overproduction of insulin will be sufficient, eliminating the need for 100% cell killing.

The receptor-targeted approach of PDT with exendin-4-IRDye700DX enables specific killing of GLP-1R–expressing cells without damaging the surrounding tissue, and the focused irradiation of the tissue of interest avoids a risk of damaging the kidneys. Because treatment of nesidioblastosis and diffuse CHI will involve irradiation of a larger part of the pancreas, there is a risk that impaired glucose tolerance will develop. However, rtPDT has advantages over near-total pancreatectomy, since it avoids the risk of exocrine pancreatic insufficiency and is much less invasive. Also, localization and quantification of the insulin-overproducing cells based on preoperative PET images using radiolabeled exendin-4 might be used for planning of the rtPDT to optimize the treatment and minimize side effects.

We believe that the data presented here, together with the advances in the technology of interstitial PDT, can provide a basis toward clinical translation of rtPDT using exendin-4-IRDye700DX. For this translation, verification of efficient targeting to human tissues, as well as the potential treatment efficacy by ex vivo analysis of human tissues, will be necessary before initiation of a first clinical trial.

## CONCLUSION

Here, we show the feasibility of rtPDT with exendin-4-IRDye700DX, in the first demonstration of efficient PDT using small molecules in vivo. In the future, ablating insulin-producing cells using rtPDT with exendin-4-IRDye700DX might provide a new, minimally invasive treatment method for patients with hyperinsulinemic hypoglycemia. Since this treatment might be applied to a specific site of the pancreas in the case of insulinomas or focal CHI or to a larger pancreatic area in the case of nesidioblastosis or diffuse CHI, it clearly has the potential to be effective to normalize blood glucose regulation in all forms of hyperinsulinemic hypoglycemia.

## DISCLOSURE

This work was supported by BetaCure (FP7/2014-2018, grant agreement 602812). Martin Gotthardt is an inventor and holder of the patent “Invention Affecting GLP-1 and Exendin” (Philips-Universität Marburg, June 17, 2009). No other potential conflict of interest relevant to this article was reported.

**KEY POINTS**

**QUESTION:** Does rtPDT with exendin-4-IRDye700DX enable effective and specific cell killing of GLP-1R–positive cells?
**PERTINENT FINDINGS:** rtPDT with exendin-4-IRDye700DX causes specific phototoxicity in GLP-1R–positive cells. The tracer accumulates in GLP-1R–positive tumors, and in vivo rtPDT causes cellular toxicity resulting in slower tumor growth.
**IMPLICATIONS FOR PATIENT CARE:** rtPDT with exendin-4-IRDye700DX might provide a new, minimally invasive treatment method for patients with hyperinsulinemic hypoglycemia.
